# Arsenic trioxide promoting ETosis in acute promyelocytic leukemia through mTOR-regulated autophagy

**DOI:** 10.1038/s41419-017-0018-3

**Published:** 2018-01-23

**Authors:** Tao Li, Ruishuang Ma, Yan Zhang, Hongdan Mo, Xiaoyan Yang, Shaoshan Hu, Lixiu Wang, Valerie A Novakovic, He Chen, Junjie Kou, Yayan Bi, Bo Yu, Shaohong Fang, Jinghua Wang, Jin Zhou, Jialan Shi

**Affiliations:** 1 Department of Hematology of the First Hospital, Harbin Medical University, Harbin, China; 2 The Key Laboratory of Myocardial Ischemia, Ministry of Education, Harbin, Heilongjiang Province China; 3 Departments of Cardiology of the Second Hospital, Harbin Medical University, Harbin, China; 4 Department of Neurosurgery of the Second Hospital, Harbin Medical University, Harbin, China; 5 Department of Cardiology of the First Hospital, Harbin Medical University, Harbin, China; 6 Department of Research, VA Boston Healthcare System, Boston, MA USA; 7 Department of Pathology, Harbin Medical University, Harbin, China; 8 Department of Hematology of the Second Hospital, Harbin Medical University, Harbin, China; 9 Department of Surgery, Brigham and Women’s Hospital, VA Boston Healthcare System, and Harvard Medical School, Boston, MA USA

## Abstract

Despite the high efficacy and safety of arsenic trioxide (ATO) in treating acute promyelocytic leukemia (APL) and eradicating APL leukemia-initiating cells (LICs), the mechanism underlying its selective cytotoxicity remains elusive. We have recently demonstrated that APL cells undergo a novel cell death program, termed ETosis, through autophagy. However, the role of ETosis in ATO-induced APL LIC eradication remains unclear. For this study, we evaluated the effects of ATO on ETosis and the contributions of drug-induced ETosis to APL LIC eradication. In NB4 cells, ATO primarily increased ETosis at moderate concentrations (0.5–0.75 μM) and stimulated apoptosis at higher doses (1.0–2.0 μM). Furthermore, ATO induced ETosis through mammalian target of rapamycin (mTOR)-dependent autophagy, which was partially regulated by reactive oxygen species. Additionally, rapamycin-enhanced ATO-induced ETosis in NB4 cells and APL cells from newly diagnosed and relapsed patients. In contrast, rapamycin had no effect on apoptosis in these cells. We also noted that PML/RARA oncoprotein was effectively cleared with this combination. Intriguingly, activation of autophagy with rapamycin-enhanced APL LIC eradication clearance by ATO in vitro and in a xenograft APL model, while inhibition of autophagy spared clonogenic cells. Our current results show that ATO exerts antileukemic effects at least partially through ETosis and targets LICs primarily through ETosis. Addition of drugs that target the ETotic pathway could be a promising therapeutic strategy to further eradicate LICs and reduce relapse.

## Introduction

Acute promyelocytic leukemia (APL) is a hematological malignancy driven by a t(15;17) chromosomal translocation that generates the promyelocytic leukemia-retinoic acid receptor (PML/RARα) fusion gene^1,2^. The prognosis for patients with APL has been revolutionized by the use of all-trans retinoic acid (ATRA) and arsenic trioxide (ATO), both of which target PML/RARα for degradation^3,4^. Recently, benefits from ATO-including therapy in APL have sparked new interest in ATO. For example, patients receiving ATO plus ATRA induction therapy experienced fewer relapses and faster complete remission compared to patients receiving standard ATRA chemotherapy^5–8^. ATO induces high rates of complete hematologic remission (CR) and molecular remission (CMR) followed by a long relapse-free survival^9^. Despite the remarkable improvement in treatment outcomes in APL, refractory and relapse remain clinically significant problems^10^. Thus, further understanding of the antileukemic mechanisms of ATO when treating newly diagnosed APL and/or relapse is urgently needed.

It is known that treatment by standard chemotherapy reagents induces apoptosis while ATRA results in differentiation^3^. However, APL relapse occurs because leukemia-initiating cells (LICs) remain untouched by conventional chemotherapy and even ATRA-monotherapy^11,12^, in contrast to ATO therapy, which implies that neither apoptosis or differentiation induction is sufficient to eradicate LICs. It is attractive to speculate whether another uncovered LIC death program exists, which can be induced by ATO. Autophagy contributes to arsenic-induced PML/RARα degradation^13^, which is responsible for LIC loss in APL cells^14,15^, and it is also widely proposed to account for arsenic-induced cell death^16–18^. However, these studies did not fully address the questions of whether or how autophagy leads to LIC death by ATO.

First described as an alternative route of bacterial killing in 2004, the formation of neutrophil extracellular traps (NETs) (ETs) is a process of cell death distinct from apoptosis, which has since been referred to as NETosis^19–21^. Formed mainly by immune cells, ETs can also be released by human leukemia cells when exposed to microorganisms, reactive oxygen species (ROS) or tunicamycin^22,23^. Studies from our laboratory have shown that APL cells from patients can also undergo this novel cell death process, producing ETs through autophagy^24,25^, that has been linked to the mechanisms of ATO. More interestingly, ATRA promotes ETosis leading to procoagulant promyelocytic extracellular chromatin^25^. However, little is known about its response to ATO treatment or the role of ETosis in leukemia cell eradication.

In this study, we characterized the concentration-dependent effects of ATO exposure on ETosis in APL cells. We also continued our previous study by investigating the upstream mammalian target of rapamycin (mTOR)-mediated autophagy pathway and the role of ROS production in this process. Finally, we explored the role of ETosis in APL LIC loss, helping identify a novel pathway to target LICs and further prevent relapse in APL patients following ATO administration.

## Results

### ATO induces ETosis and apoptosis in NB4 cells in a dose-dependent manner

**Fig. 1:**
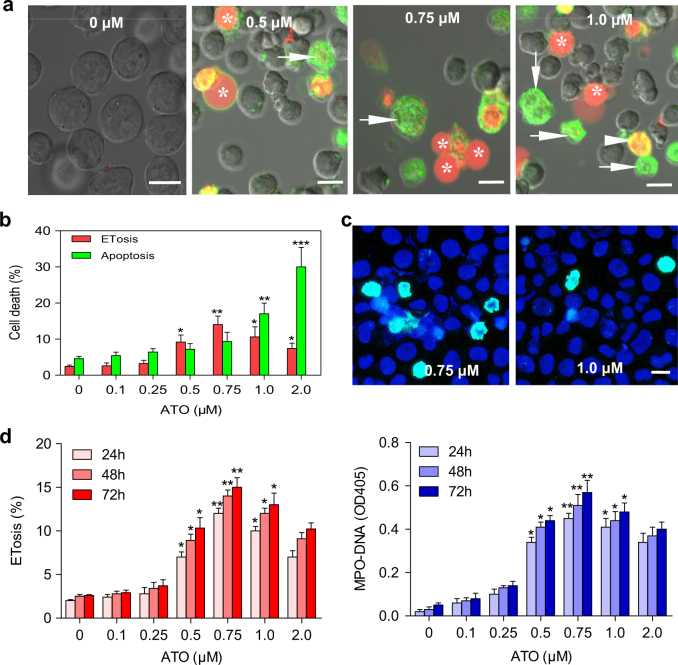
ATO induces ETosis and apoptosis in NB4 cells in a dose-dependent manner **a** NB4 cells were incubated in 0, 0.1, 0.5, 0.75, 1.0, or 2.0 μM ATO for 48 h and then stained with lactadherin (green) and PI (red). Nuclei bubbled, lost shape, expanded, and filled most of the cytoplasm, enclosed by a thin layer of membrane in ETotic cells (*). In contrast, cells undergoing early apoptosis showed diffuse rim staining by lactadherin but no PI staining (arrow). Late apoptotic cells were stained brightly with lactadherin (green) showing typical condensed nuclei (arrowhead). **b** Cells undergoing ETosis or apoptosis were counted and analyzed as described in methods (*n* = 5). **c** Immunofluorescence analysis of ET components. After incubation with 0.75 μM (left) or 1.0 μM (right) ATO for 48 h, NB4 cells were stained with DAPI (blue) and anti-histone-3 (green). **d** Quantification of the percentage of ETotic cells and the concentration of MPO–DNA complexes levels in the supernatant showed dose-dependent and time-dependent fluctuation (*n* = 5). All values are mean ± SD. *P < 0.05, **P < 0.01 and ***P < 0.001 vs. 0 (PBS) μM. Bars represent 15 μm in **a**,** c**

To distinguish the effect of ATO on ETosis and apoptosis, lactadherin and propidium iodide (PI) were used to stain NB4 cells^24,25^. In ETotic cells, the chromatin expands while the cytoplasmic membrane remains intact. PI staining can be observed in the absence of lactadherin membrane staining (green) or visible membrane blebbing. Cells undergoing ETosis could be seen releasing a single swelling bubble that stained with PI^24,25^. To investigate the effect of varying concentrations of ATO on ETosis in cultured NB4 cells, an APL cell line, cells were treated with 0, 0.1, 0.25, 0.5, 0.75, 1.0, or 2.0 μM ATO for different time points. When cultured for 48 h, concentrations of ATO over 0.5 μM caused a significant increase in the number of ETotic cells (Fig. 1a, b). When NB4 cells were treated with ATO at 1.0 μM or higher concentrations, both ETotic and apoptotic cells were visible (Fig. 1a). Using immunofluorescence, we identified that promyelocytic ET backbone as DNA-histone (Fig. 1c). ETosis% counted by DAPI/anti-histone-3 and lactadherin/PI staining were comparable (Supplementary Fig. S1)^24^. PI staining was used to define the morphological changes leading to ETosis. Evaluating the changes in nuclear size and shape, we identified four morphologies (Supplementary Fig. S2): (i) round, (ii) budding, (iii) spread nuclei, and (iv) extracellular DNA. Because DNA material ultimately became cell-free, MPO–DNA complexes could be detected in the supernatant and used as a marker of ETosis^26^. Our results showed that ETosis dramatically increased at 0.5 μM, peaked at 0.75 μM, and decreased at higher ATO concentrations (Fig. 1d).

### Moderate concentrations of ATO mainly promotes ETotic cell death in NB4 cells

**Fig. 2:**
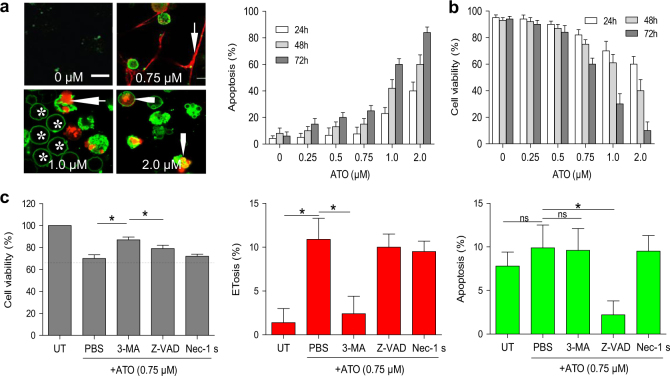
ATO induces ETotic cell death in NB4 cells **a** NB4 cells were treated with 0 (PBS), 0.25, 0.5, 0.75, 1.0, or 2.0 μM ATO for 48 h and then stained with lactadherin (green) and PI (red). Confocal microscopy images showed that untreated NB4 cells showed little staining with either lactadherin or PI. The nuclei of ET-releasing cells spread into the extracellular space (arrow). NB4 cells undergoing early apoptosis showed ring-like staining by lactadherin but no PI staining (*). Cells in late apoptosis had nuclei that were condensed and staining by both lactadherin and PI (arrowhead). Apoptosis was determined by lactadherin/PI staining for 15 min at room temperature analyzed by flow cytometry. The data represents mean ± SD of three independent experiments done in duplicate. **b** Cell viability was detected by trypan blue exclusion. The data show mean ± SD of three independent experiments done in duplicate (*n* = 3). **c** NB4 cells were preincubated with corresponding cell death inhibitors (Z-VAD, Nec-1, 3-MA) for 1 h, and treated with 0.75 μM ATO for 48 h. The cell viability in untreated group was counted as 100%. Apoptosis was analyzed by flow cytometry and ETosis were counted as described in methods (*n* = 5). All values are mean ± SD. *P < 0.05, ns = not significant. Bars represent 10 μm in **a**

To better understand the mechanism behind ATO-induced cell death, we dual stained cells with lactadherin and PI (Fig. 2a). Apoptosis was analyzed by flow cytometry, and confirmed by frequency of cells that were TUNEL-positive. Surprisingly, the results showed that the percentage of apoptotic cells (lactadherin^+^ and PI^+^) had a marked increase only at concentrations over 1.0 μM (Fig. 2a), while the viability of NB4 cells dramatically decreased at 0.75 μM ATO (Fig. 2b). Thus, 0.75 μM ATO treatment probably induced non-apoptotic cell death. To confirm the role of ETosis in non-apoptotic cell death induced by 0.75 μM ATO, NB4 cells were preincubated with various cell death inhibitors. We found that 3-methyladenine (3-MA) significantly increased the number of viable cells reduced by ATO, while other cell death inhibitors had few effects. Moreover, 3-MA only decreased ETosis induced by ATO while having limited effects on apoptosis (Fig. 2c). Taken together, these results suggested that the majority of NB4 cell death induced by 0.75 μM ATO was ETosis.

### ATO increase mTOR signaling-dependent autophagy that mediated ETosis

**Fig. 3:**
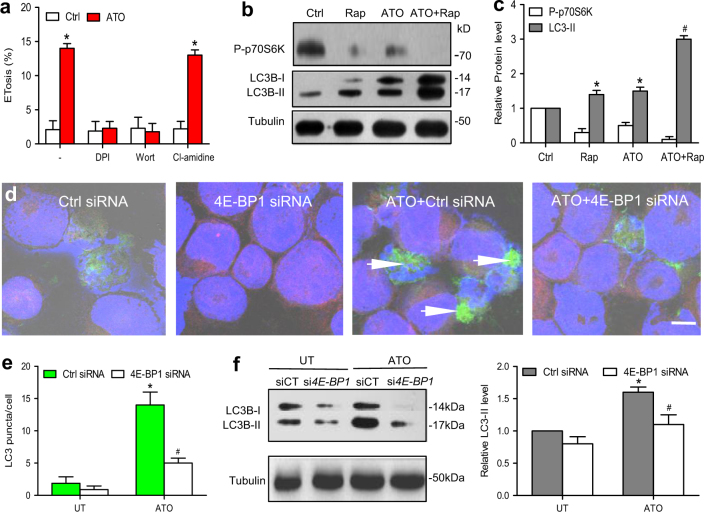
ATO induce autophagy through the mTOR pathway **a** NB4 cells were preincubated with the indicated inhibitors (pretreatment with DPI (10 μM, 4 h), wortmannin (1 mg/ml) for 30 min, Cl-amidine (200 μM)) before treatment with ATO (0.75 μM, 48 h) to induce ETosis. **b**, **c** NB4 cells were treated with vehicle or rapamycin (10 nM) in the presence or absence of ATO (0.75 μM) for 48 h. Cell lysates were examined by western blot by the use of anti-P-p70S6K, anti-LC3, or anti-tubulin antibodies. Quantification of the data shown in panel **c** (*n* = 3). *P < 0.05 vs. control. **d** NB4 cells were stained with DAPI (blue) and anti-LC3 (green). Immunofluorescence images showed inhibition of the negative downstream effector of mTOR pathway by knocking down *4E-BP1* reduced the aggregation of LC3 (green). **e** The LC3 puncta per cell in ATO-treated NB4 cells measured by immunofluorescence (*n* = 3). **f** The levels of LC3B-I and LC3B-II were detected by western blotting (*n* = 3). All values are mean ± SD. *P < 0.05 vs. untreated group, ^#^*P* < 0.05 vs. control **e**, **f**. Bars represent 20 μm in **d**. DPI diphenyleneiodonium chloride, Wort Wortmannin, Rap rapamycin (mTOR inhibitor), PAD4 peptidylarginine deiminase 4, UT untreated group, Ctrl, control

The ETotic process involves interplay between autophagy, the cytoskeleton, superoxide production, and histone citrullination^21,27^. To further unravel the molecular mechanism by which ATO primes cells for ETosis, we investigated possible signaling pathways preceding ETosis. Diphenyleneiodonium chloride (DPI) and wortmannin but not Cl-amidine were able to block ATO-triggered ETosis (Fig. 3a). When nutrient deprivation is used to induce autophagy, mTOR-mediated phosphorylation of p70 ribosomal S6 kinase (p70S6K) is prevented. Therefore, a reduced level of phosphorylated p70S6K (P-p70S6K) can be used as an indicator of mTOR-dependent autophagy^28^. ATO-treated NB4 cells exhibited increased levels of LC3-II and decreased levels of P-p70S6K by immunoblotting (Fig. 3b, c), suggesting that ATO activated autophagy via the mTOR pathway. Moreover, siRNA-mediated inhibition of the negative downstream effector of mTOR pathway, translational repressor 4E-BP1 (Supplementary Fig. S3), reversed the effects of ATO on LC3-positive structures (autophagosomes) (Fig. 3d, e), LC3 expression (measured by western blot) and ETosis (Supplementary Fig. S4), confirming ATO-induced ETosis was dependent on mTOR-mediated autophagy.

### Rapamycin enhances the autophagy-dependent ETosis but not autophagy-independent apoptosis in response to ATO

**Fig. 4:**
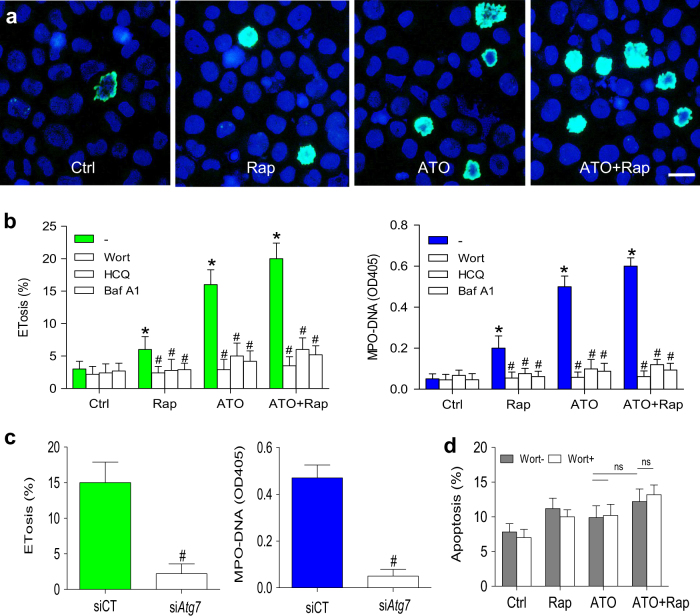
Rapamycin enhances the effects of ATO on autophagy-dependent ETosis **a**,** b** NB4 cells were cultured in rapamycin (10 nM) or/and ATO (0.75 μM) for 48 h and treated with vehicle or with various autophagy inhibitors (wortmannin, HCQ and Baf A1). **a** NB4 cells were stained with DAPI (blue) and anti-histone-3 (green). Immunostaining images showed ATO-induced ETosis was enhanced by concomitant addition of rapamycin. **b** Quantification of the percentage of ETotic cells and corresponding MPO–DNA complexes concentrations for the different treatments (*n* = 5). **c** NB4 cells were transiently transfected with *Atg7* siRNA (si*Atg7*) at a concentration of 100 nM and scrambled siRNA (scr) was used as a negative control (siCT). Seventy-two hours after transfection, cells were treated with ATO (0.75 μM) for 48 h. The percentage of ETosis and the concentration of MPO–DNA complexes were then measured (*n* = 5). **d** Apoptosis was analyzed by flow cytometry (*n* = 5). All values are mean ± SD. *P < 0.05 vs. control, ^#^*P < *0.05 vs. Wort-, ns = not significant. Bars represent 20 μm in **a**. UT untreated group, Rap rapamycin, Wort wortmannin

Because induction of autophagy increase ETosis^24^ and the aforementioned experiments demonstrated a role of mTOR in ATO-induced autophagy, we also examined whether downregulation of mTOR activity by rapamycin would affect ETosis in NB4 cells. We found that rapamycin alone was capable of inducing a modest increase ETosis and that this drug potentiated ATO-mediated ETosis (Fig. 4a, b). However, the same combination treatment (ATO + rapamycin) of NB4 cells along with inhibition of autophagy with various autophagy inhibitors (wortmannin, hydroxychloroquine (HCQ) and bafilomycin A1 (Baf A1)) resulted in a significant decrease in ETosis (Fig. 4b). Furthermore, we knocked down autophagy-related gene *Atg7* using small-interfering RNA (siRNA) to confirm the role of autophagy in ATO-induced ETosis in APL. As shown in our previous work^24^, inhibition of autophagy nearly abolished autophagy as the markedly decreased conversion of LC3B-I to LC3B-II. Knocking down of *Atg7* resulted in significantly reduced ATO-induced ETosis and MPO–DNA complexes (Fig. 4c). Interestingly, rapamycin did not increase ATO-induced apoptosis, which was not affected by autophagy inhibition with wortmannin (Fig. 4d).

### The role of ROS production during ETosis

We monitored intracellular ROS production in NB4 cells to investigate its role in ETosis. The results showed that intracellular ROS was increased in NB4 cells activated by ATO, and remained at baseline if inhibited by NADPH oxidase (Nox) with DPI (Supplementary Fig. S5). Inhibition of autophagy with wortmannin or activation of autophagy with rapamycin did not alter basal ROS levels or ATO-induced ROS production (Supplementary Fig. S6). We then assessed whether ROS was essential for ETosis. NB4 cells were stained with DAPI and anti-histone-3 to identify ETotic cells. While ATO notably increased histone staining, the addition of DPI abrogated ETosis (Supplementary Fig. S5). Overall, these results demonstrated that Nox-dependent ROS production plays a key role as an early event of ATO-induced ETosis, independent of autophagy.

### Rapamycin enhances ATO-induced ETosis in NB4 cell lines and APL cells from patients at diagnose and after relapse

**Fig. 5:**
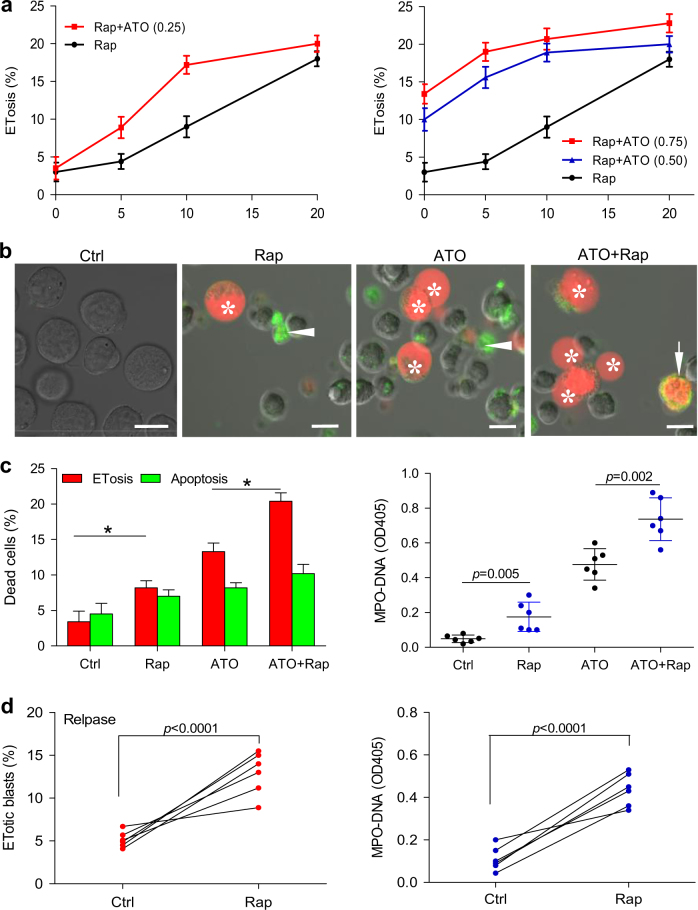
Effects of ATO and rapamycin on apoptosis and ETosis in NB4 cells and patient APL cells **a** ETosis induced by low doses of ATO (0.25, 0.5, 0.75 μM) and increasing concentrations of rapamycin (0, 5, 10, and 20 nM) was evaluated at 48 h in NB4 cells. Values are expressed as mean ± SD (*n* = 6). **b**,** c** APL cells from newly diagnosed APL patients were treated with vehicle or rapamycin (10 nM) in the presence or absence of ATO (0.75 μM) for 48 h. **b** APL cells were stained with lactadherin (green) and PI (red) and imaged with confocal microscopy. ETotic cells showed nuclei (stained with PI) that had lost shape and expanded to fill most of the cytoplasm, but did not stain with lactadherin (*). In contrast, cells undergoing early apoptosis showed diffuse rim staining by lactadherin but no PI staining (arrowhead). Late apoptotic cells had condensed nuclei and were stained by both lactadherin and PI (arrow). **c** Cells undergoing ETosis or apoptosis were counted as described in methods (*n* = 6). MPO–DNA complexes levels were also measured (*n* = 6), *P < 0.05. **d** Primary APL cells from patients at relapse were treated with vehicle or rapamycin (10 nM) in the presence of ATO (0.75 μM, 48 h). The percentage of ET-formation blasts and MPO–DNA complexes in the supernatant were assayed in duplicate wells (*n* = 6). Bars represent 15 μm in **a**. Rap rapamycin

For the evaluation of the combinated effects on ETosis, NB4 cell line was first treated with a fixed non-toxic concentration of ATO (0.25 μM, 48 h) and graded concentrations of rapamycin. The drug combination resulted in synergistic effects at all combination data points. When cells were treated with toxic concentration of ATO (0.5 μM and 0.75 μM, 48 h) and graded concentrations of rapamycin, lower doses of rapamycin resulted in synergistic effects while higher doses of rapamycin (10 and 20 nM) suggested additive effects (Fig. 5a).

Since resistance is the major puzzle in APL therapy, we investigated whether this combination (ATO + rapamycin) is effective on ATRA-resistant and ATO-resistant cell lines (NB4-R4 and NB4 ATO-R). Increasing doses of rapamycin were tested, starting at 5 nM and scaling up to 20 nM. Marked increase of ETosis was initially detected NB4-R4 cells using 10 nM rapamycin, whereas 20 nM rapamycin induced a significant increase in ETosis not only in NB4-R4 but also in NB4 ATO-R cells. In NB4 ATO-R cell line, less sensitive to rapamycin as single agent, the combination of 1 μM ATO with rapamycin (20 nM) resulted in a remarkable increase in the percentage of ETotic cells, as shown by lactadherin/PI staining after 48 h (Supplementary Fig. S7).

To confirm the effect of rapamycin and ATO on ETosis, primary APL blasts from newly diagnosed APL patients (patient 1–6 in Table 1) were incubated with rapamycin (10 nM) in the presence or absence of ATO. We found that rapamycin alone was capable of inducing a modest increase (from 3.4 to 8.2%) in ETosis and that this drug potentiated ATO-mediated ETosis (from 13.3 to 20.4%) (Fig. 5b, c), suggesting that the effects of ATO and rapamycin are additive. Furthermore, addition of the mTOR inhibitor rapamycin strongly enhanced the percentage of ATO-mediated ETosis (paired *P*-value < 0.0001, *n* = 6) in APL cells taken from patients after relapse (patient 7–12 in Table 1) (Fig. 5d). Measurements of MPO–DNA complexes in the supernatant were used to indirectly quantify ETs. We found that the increase in ETotic APL cells was paralleled by an elevated abundance of MPO–DNA complexes in the supernatant (Fig. 5b, c).

**Table 1:** APL patients’ characteristics and results of ATO therapy

No.	Sex/age	Diagnosis	Blasts (BM%)	WBC (×10^9^/l)	Plts (×10^9^/l)	Hb (g/l)	Outcome
1	F/44	M3/bcr1	80	5.1	43	62	HCR
2	F/45	M3/bcr1	84	10.4	27	57	HCR
3	M/34	M3/bcr3	78	4.5	37	77	HCR
4	M/38	M3/bcr1	88	11.2	33	68	HCR
5	F/17	M3/bcr3	88	28.2	26	90	HCR
6	F/44	M3/bcr1	85	18.2	45	67	HCR
7	F/42	M3/bcr1	95	25.7	31	77	PR
8	M/41	M3/bcr1	92	26.1	22	60	PR
9	M/45	M3/bcr1	87	14.2	14	77	HCR
10	F/34	M3/bcr1	90	32.5	12	79	HCR
11	M/53	M3/bcr3	92	20.4	77	58	PR
12	F/29	M3/bcr1	91	24.4	34	86	HCR
13	M/39	M3/bcr3	94	6.2	37	66	HCR
14	M/35	M3/bcr3	94	26.2	33	76	HCR
15	M/62	M3/bcr1	80	4.5	55	76	HCR
16	M/23	M3/bcr1	89	4.1	12	62	HCR
17	M/33	M3/bcr1	82	20.2	13	61	ED
18	M/23	M3/bcr3	81	18.9	19	62	HCR
19	F/39	M3/bcr3	83	17.7	12	68	HCR
20	F/41	M3/bcr1	87	12.8	32	55	PR
Ref. range			0–0.4	4–10	100–300	110–170	HCR

The main clinical and laboratory features of 20 newly diagnosed APL patients and results of ATO therapy during induction therapy

*Blasts* promyelocytes + blasts, *PB* peripheral blood, *WBC* white blood cell, *Plts* platelets, *Hb* hemoglobin, *bcr* breakpoint cluster region (bcr1 = intron 6, bcr3 = intron 3), *HCR* complete hematologic remission, *PR* partial remission, ED early death

### Combined rapamycin and ATO treatment synergistically decreases LIC production

**Fig. 6:**
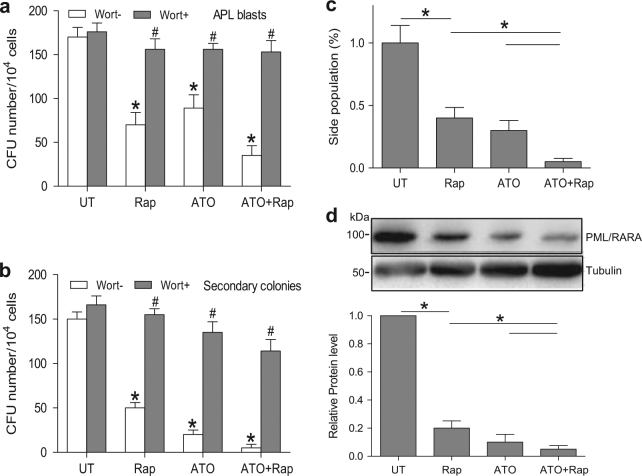
ETosis contributes to LICs loss in APL in vitro **a** Primary APL cells isolated from patients were cultured in methylcellulose for a week with rapamycin (10 nM) or ATO (0.75 μM) alone or in combination and in the presence or absence of wortmannin (1 mg/ml). Number of colonies is shown (*n* = 8). **b** After 1 week of treatment, cells were harvested, washed and cultured in semisolid medium in the absence of any drug (*n* = 8). Number of secondary colonies is shown. *P < 0.05 vs. UT, ^#^*P < *0.05 vs. wortmannin (-). **c**,** d** NB4 cells treated with rapamycin (10 nM) and/or ATO (0.75 μM) for 48 h. **c** NB4 side population was defined by exclusion of Hoechst 33342 in the absence or presence of verapamil. The percentage of cells representing the side population was measured. **d** Lysates from NB4 cells were analyzed by immunoblotting by the use of antibodies against anti-PML/RARA, and tubulin was used as a loading control (*n* = 5), *P < 0.05. Wort, wortmannin

Primary APL blasts were isolated from APL patients (patient 13–20 in Table 1) and cultured in methylcellulose, a semisolid medium, in the presence of rapamycin, ATO, or both. ATO resulted in a striking decrease in colony-forming units when in combination with rapamycin compared with either drug alone (Fig. 6a). Furthermore, when cultured for an additional week, treated colonies had a reduction in the number of secondary colonies (Fig. 6b). Thus, rapamycin and ATO synergistically diminished the clonogenicity of APL cells. Meanwhile, inhibition of ETosis with wortmannin blocked the effects of both ATO and rapamycin (Fig. 6a, b). To confirm our results obtained from primary APL cells, we investigated the effect of ATO-induced ETosis on the side population of NB4 cells, a model of APL-derived LICs. We observed that rapamycin exhibits synergism with ATO (0.75 μM, 48 h) for efficient side population clearance (Fig. 6c) and PML/RARα degradation in NB4 cells (Fig. 6d).

### Combination of rapamycin and ATO reduces leukemic burden and LICs in an APL model

**Fig. 7:**
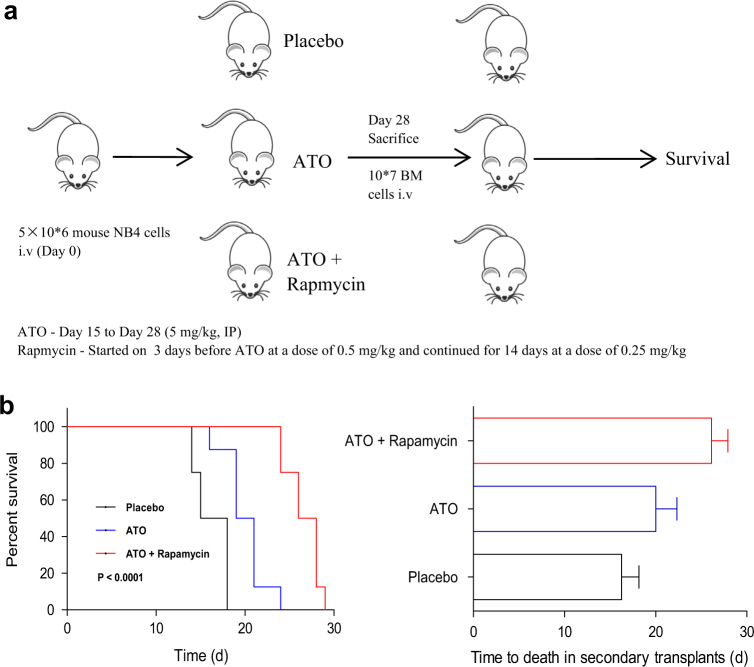
ETosis contributes to LICs loss in in an APL model **a** Schematic representation of secondary transplantation experiment (not treated post transplantation). Mice were then observed up to their death. **b** The survival curve and median survival time (days) of the secondary transplantation of bone marrow cells from the three animal treatment groups. Statistical significance was calculated using log-rank test and nonparametric, unpaired, two-tailed, Mann–Whitney test. BM bone marrow, IP intraperitoneal injection, i.v. intravenous injection. Values are mean ± SD (*n* = 8). UT untreated group

After identifying the effect of ATO-induced ETosis on the side population of NB4 cells, we next confirmed the combination’s effect on LICs in a xenograft APL model using NB4 cell line. The combination therapy showed a significant reduction in the tumor burden on day 28 compared with mice treated with either agent alone, as evidenced by reduction in spleen size, decreased PML-RARA copy numbers and decreased bone marrow blasts analyzed by flow cytometry and immunohistochemistry (hematoxylin and eosin staining (data not show)). We then performed secondary transplantation of the bone marrow cells of leukemic mice harvested on day 28 after treatment (Fig. 7a). We observed that ATO alone or ATO + rapamycin produced significantly longer survival of secondary recipients. Mice receiving bone marrow treated with ATO + rapamycin had a median survival time of 27 days compared to 16.5 days with placebo or 20 days with ATO alone (Fig. 7b). Altogether, these data suggest that this combination is able to significantly decrease the LIC compartment in a mouse model of APL.

## Discussion

This is the first study to show that ATO significantly increases the amount of ETosis in APL cells. ATO has dose-dependent antileukemic effects on NB4 cells, preferentially inducing ETosis at moderate concentration (0.5–0.75 μM) and switching to apoptosis at relatively high doses (1.0–2.0 μM). Further investigation indicated that ATO-induced ETosis requires both mTOR-mediated autophagy and Nox-dependent ROS production. More importantly, we found that rapamycin promoted ATO-induced ETosis leading to decreased LIC activity, suggesting that ETosis facilitates LIC loss in APL cells. These results further clarify the cellular and molecular mechanisms of ATO treatment. Our discovery suggests that therapy-triggered ETosis could be used to target LICs and, therefore, reduce relapse risk in patients.

We have recently found that ATRA promotes a novel cell death process, named ETosis, in APL cells^24,25^. Since ATO exerts dose-dependent cellular effects^16,17,29^, we investigated its dosage effect on ETosis. In concordance with the finding that low doses of ATO cause only minimal cell death^29^, ETotic cell death started and peaked at median doses of ATO. ATO at high concentrations triggered the apoptosis pathway^29^, which is thought to block the ETosis pathway^20^. As expected, we observed that ETosis decreased at higher concentrations. We performed all our experiments in the presence APL serum containing cytokines, which mimicked the conditions at inflammatory sites as a two-hit model to favor ET formation^30^. It is not surprising, however, that ATO can induce this novel form of cell death. Indeed, previous reports have shown that ATO is a potent inducer of apoptotic and non-apoptotic cell death^16,17^. While our work strongly supports a role for ATO in priming ETosis, the fluctuating levels of circulating APL-derived ETs in the plasma of hospitalized patients treated with ATO need to be explored. An assay of patient plasma for MPO–DNA complexes, a marker of ETosis, could potentially be used to predict drug sensitivity and/or risk of relapse.

The process of ETosis is distinct from apoptosis, in that there is no caspase activation or phosphatidylserine (PS) exposure before cell death, and the morphological characteristics of the two forms of active cell death are different^24,25^. Beyond apoptosis, ATO has at least a partial tendency to favor ETosis which results in lower overall PS exposure on APL cells. PS exposure is a major mechanism through which APL cells support procoagulant activity^31^. Therefore, our results explain the mechanism by which ATO treatment results in rapid correction of patient coagulopathy preceding disappearance of the circulating leukemic promyelocytes^32^. Thus, this alternative cell death process may represent a therapeutic target in the treatment of resistant cases and attenuating apoptosis-associated complications. NET-like structures are also generated in other myeloid cell lines^23^, and ATO is known to exert anticancer effects on other hematopoietic tumor cells^17,18^. Further studies are needed to determined whether ATO induces ETosis in other hematological malignancies.

ATO induces autophagic cell death in several types of tumor cells^16,17^, and degrades PML/RARα protein via mTOR pathway-mediated autophagy in APL cells^13^. In this study, we have identified the pivotal role for the mTOR pathway in ETosis via regulation of autophagy. From a novel cell death perspective, our data explains the results obtained by Altman et al.^33^ that pharmacological inhibition of mTOR enhances the suppressive effects of ATO on leukemic progenitor colony formation. Beyond the prior reports, we found that mTOR-mediated signals regulated the ETotic response to ATO implying that mTOR could be a potential pathway to suppress LIC activity. Although the role of the PI3K-Akt-mTOR pathway in NET formation is contradictory, reports of fMLP-induced NETosis are consistent with our results showing that this pathway inhibits autophagy^34^. Our results reveal a new facet of this pathway, suggesting that ETosis might be directly linked to, or be a variant of, the autophagic cell death pathway. This preclinical data suggests the need for a clinical investigation of ATO in combination with other agents interfering with mTOR-mediated autophagy. Combined therapy could contribute to the killing of APL LICs through the induction of ETosis and thereby improve remission rates.

Oxidative production is one of the proposed mechanisms by which ATO affects cellular behavior. NB4 cells exposed to ATO show increased Nox-derived ROS production^35–37^, which shapes a critical component of NETosis^27^. In accordance with other studies, our results show that ATO enhanced ET formation via a Nox-dependent mechanism, which is supported by the finding that Nox is involved in ATO-induced cytotoxicity^35^. Evidence has emerged that autophagy and activated Nox are both required in ETosis where they work together to inactivate caspases and prevent cells from entering apoptosis^26,38,39^. This explains our observation that blocking either autophagy or ROS generation decreases ETosis in NB4 cells. Based on the current findings, it seems that ROS regulate autophagy that mediates ATO-induced ETosis^40^.

Since ATO-based therapy is strongly correlated with APL LIC eradication^12,41^, we tested whether ATO-induced ETosis plays a role in LIC loss. Rapamycin as a classic autophagy inducer augmented ATO-induced cytotoxicity in NB4 cells^42^. Since LICs remain untouched by apoptosis-inducing conventional chemotherapy^11,43^ and rapamycin did not promotes apoptosis by ATO, it is convincing that activation of the ETotic rather than apoptotic cell death pathway contributes to rapamycin-enhanced LIC loss by ATO. In addition, we confirmed the role of ETosis in ATO-induced APL LIC loss through inhibition experiments. Therefore, rapamycin potentiates ATO-induced LIC loss in patient APL cells and stem cell population clearance in NB4 cells at least partially through ETosis. Based on these encouraging in vitro results, we further validated the efficacy of the combination of rapamycin and ATO in a transplantable mouse model of APL.

At the molecular level, a previous study suggested that autophagy contributes to ATO-induced degradation of the PML/RARα^13^, whose degradation is necessary for LIC eradication^14,43^. By monitoring APL LIC activity-linked oncoprotein PML/RARα, we observed that enhanced ETosis significantly correlated with oncoprotein degradation. Thus, it seems that autophagic degradation of PML/RARA leads LICs to ETosis. This study goes beyond the prior reports, correlating the cellular fate of LICs with ETotic cell death. Besides loss of self-renewal, differentiation and apoptosis, our finding adds ETosis to the potential cellular mechanisms of LIC clearance, though there is not yet direct evidence of ETotic LICs.

Since prompt treatment with ATO has been the most important step in treating and preventing relapse, future therapeutic strategies could focus on combined application of rapamycin or autophagy inducer with ATO to eradicate LICs and further decrease risk of relapse, especially in high-risk APL patients. In brief, our study uncovers the role of ETosis in LIC eradication, representing a novel mechanism for ATO anticancer activity. Thus, enhancing ATO-induced ETosis could be a potential strategy to eradicate LICs.

## Materials and methods

See Supplementary files for a full description of materials and methods (available on the Cell Death and Disease Web site).

### Reagents

ATO, rapamycin (200 μM stock in EtOH), 5′-(4-Fluorosulfonylbenzoyl) adenosine hydrochloride (wortmannin), 3-MA (stock 100 mM in EBSS), HCQ and Baf A1 (stock 200 μM EtOH) were from Sigma-Aldrich (St Louis, MO). ATO stock solutions were made at the concentration of 1 mM with phosphate-buffered saline (PBS) and diluted to working concentrations before use.

### Patients

Twenty newly diagnosed APL patients admitted to the First and Second Affiliated Hospital of Harbin Medical University from May 2014–February 2016 agreed to participate in the study after informed consent. The diagnosis was based on clinical data, morphology, cytochemistry, immunology, cytogenetics, and molecular pathology testing or alternatively confirmation of the presence of the t(15;17) (PML-RARα) fusion gene^31^. Compassionate use of ATO was initiated in all the 20 previously untreated APL patients, and this treatment was very well tolerated. This study was approved by Ethics Committee of Harbin Medical University and conducted in accordance with the Declaration of Helsinki.

### Cell lines and primary cells

Human APL NB4 cell line and ATRA-resistant cell line, NB4-R4, were gifts from Dr. James O’kelly (Los Angeles, CA). While the ATO-resistant cell line, NB4-ATO-R, was established by culturing in increasing concentrations of ATO, with initial concentration of 200 nM, until the cell line survived a concentration of 1 μM. The resistant cell line was then maintained in complete RPMI 1640 medium containing 1 μM ATO. NB4 cells transiently transfected with *Atg7* siRNA were obtained in the same way as our previous work^24^. Bone marrow samples from APL patients were collected before intravenous ATO (0.1 mg/kg per day) treatment and at hematological relapse after obtaining a written informed consent. APL cells were harvested from the bone marrow of 14 newly diagnosed APL patients for further experiment. APL cells from the BM of another six APL patients at hematological relapse were treated with vehicle or rapamycin (10 nM) in ATO (0.75 μM) for 48 h.

### Cell culture

Freshly isolated APL blasts were obtained from bone marrow specimens by centrifugation through Ficoll-Hypaque and were used for experiments immediately. These cells and NB4 cells (5 × 10^5^/ml) were resuspended in complete RPMI 1640 medium supplemented with 10% FBS, 10% APL serum, 2 mM L-glutamine and 1% penicillin–streptomycin solution at 37 °C in a 5% CO_2_ humidified atmosphere.

### Confocal microscopy

To characterize cell death, smears of APL or NB4 cells (5 × 10^5^) were incubated with DAPI (100 ng/ml)^44^ or the indicated concentration of PI and FITC-labeled lactadherin^45^. Cells were washed to remove unbound proteins and analyzed immediately on Zeiss LSM 510 Meta confocal microscope (Carl Zeiss Jena GmbH, Jena, Germany). The samples were excited with the 488 nm emission line of a krypton-argon laser, and the emission wavelength overlap was restricted with narrow bandpass filters.

### Determination of ETosis and apoptosis

Cells undergoing ETosis were characterized by rounded morphology, PI staining, and the presence of nuclear content diffused throughout the cell^24,25^. When cells were stained with DAPI/anti-histone-3, ETosis was identified by histone-3-stained cells^24^. When cells were stained with lactadherin and PI, cells in early apoptosis showed diffuse rim staining by lactadherin but no PI staining. Late apoptosis was characterized by condensed nuclei and stained by both lactadherin and PI. ^45^ Apoptosis with PI staining was defined by the presence of membrane blebbing and nuclei fragmentation. Cells were counted from six random fields in triplicate wells for each condition and expressed as percentage of total number of cells in the field^46^.

### Quantification of ETosis

To quantify ETs in cell culture supernatant, a capture ELISA to measure myeloperoxidase (MPO) associated with DNA was performed as described previously^26,47^. For the capture antibody, Mouse MPO ELISA kit (Hycult biotech, HK210–01) was used according to the manufacturer’s directions. A peroxidase-labeled anti-DNA mAb (component No.2, Cell Death ELISAPLUS, Roche; Cat. No: 11774424001) was used.

### Cell apoptosis assay

For the apoptosis assay, cells were washed with PBS, resuspended in binding buffer from BD Biosciences and stained with Lactadherin-FITC and PI for 15 min. Fluorescence was measured with a flow cytometer, and the membrane integrity of the cells was simultaneously assessed by the PI exclusion method. Apoptosis was determined using the Lactadherin-FITC apoptosis detection kit (BD Pharmingen, San Diego, USA), according to the manufacturer’s instructions. Prepared cells were analyzed with FACScan flow cytometer and CELLQuest software (Becton Dickinson, Franklin Lakes, USA). The TUNEL (terminal deoxynucleotidyl transferasemediated deoxyuridine triphosphate nick end labeling) assay was performed using the in situ cell death detection kit (Roche, Philadelphia, USA), according to the manufacturer’s instructions.

### Inhibition assays

For autophagy inhibition assays, cells were pretreated with the autophagy inhibitors wortmannin (1 mg/ml) for 30 min^24^, 3-MA (5 mM) during the last 24 h of the incubation, HCQ (20 μM) or Baf A1 (100 nM) during the last 12 h of the incubation^13,48^. Cells were pretreated with DPI (10 μM, 4 h) to inhibit NADPH oxidase, and cultured with peptidylarginine deiminase 4 (PAD4) inhibitor Cl-amidine (200 μM) to block histone citrullination, necrostain-1 (Nec-1 s) (30 μM) to inhibit necroptosis, or z-VAD-FMK (25 μM) to inhibit apoptosis.

### Assays of primary hematopoietic progenitors from APL patients

To evaluate drug effects on LIC activity in APL cells, APL cells were cultured (10,000 cells per well) in the methylcellulose medium, supplemented with recombinant cytokines (GF M3434, StemCell Technologies) in the presence or absence of the drugs. Rapamycin (10 nM) was used alone or in combination with ATO (0.75 μM). After a week of treatment, colonies were counted and cells were harvested, washed and, when specified, plated in the methylcellulose medium for a further week without drugs.

### Side population of NB4 cells

In cell lines, the stem cell population is contained in the so-called side population determined by staining exclusion in the presence or absence of ABC transporter inhibitors such as verapamil^49,50^.

### Secondary transplantation experiments

In all, 5 × 10^6^ NB4 cells were injected into 6–8-weeks-old SCID mice followed by treatment with ATO with or without rapamycin or no drug treatment (placebo) from day 15–day 28. On day 28, the mice were sacrificed and 1 × 10^7^ bone marrow cells were transplanted into a new batch of 6–8-weeks-old mice (no treatment post-transplant) and followed up to their death. Details of methodology used for “experimental animals”, “in vivo xenograft model”, and “secondary transplantation experimental design” are provided in “Supplemental Methods”.

### Statistical analysis

The results are expressed as mean ± standard deviation (SD), and all statistical analyses were performed using Student’s *t*-test (two-tailed, unpaired). A *P*-value of 0.05 or less was considered significant. In cell viability assays, the data are normalized to control cells and expressed as the percentage of live cells relative to total cells.

## Electronic supplementary material

Supplementary Materials and Methods

Supplementary Results
