# Supplementary material for: Arsenic trioxide promoting ETosis in acute promyelocytic leukemia through mTOR-regulated autophagy
Source: Cell Death Dis. 2018 Jan 23;9(2):75. doi: 10.1038/s41419-017-0018-3 (PMC5833714; doi:10.1038/s41419-017-0018-3)
Supplement: Supplementary file 1 — Supplementary Materials and Methods [file 41419_2017_18_MOESM1_ESM.docx]

**Supplementary Materials and Methods:**

**Reagents**

RPMI 1640 medium and fetal bovine serum (FBS) were obtained from Gibco (Grand Island, NY, USA). Ficoll-Hypaque, poly-d-lysine, bovine serum albumin (BSA), 4',6-diamidino-2-phenylindole (DAPI), pan-caspase inhibitor z-VAD-FMK (V116-2MG) and diphenyleneiodonium chloride (DPI) were all from Sigma-Aldrich (St Louis, MO, USA). Cl-amidine was from Cayman (Shanghai, China). Necroptosis inhibitor necrostain-1 (Nec-1 s, 2263-1) was from BioVision (Milpitas, CA, USA). Propidium iodide (PI) was from Shanghai DobioCO, LTD (Shanghai, China). siRNA against *4E-BP1* (sc-29595) and antibodies against 4E-BP1were obtained from Seebio (Shanghai, China).

**Antibodies**

FITC-lactadherin was obtained from Haematologic Technologies Inc. (Burlington, VT, USA). Rabbit anti-histone 3-Alexa Fluor 488 mAb (ab154206), anti-β-tubulin (ab6046) and anti-PML+RARα (ab43152) were from Abcam (Cambridge, MA, USA). Rabbit anti-LC3α/β polyclonal Ab (sc-292354) was obtained from Santa Cruz Biotechnology Inc. (Santa Cruz, CA, USA). Goat anti-rabbit Cy3 antibody (Invitrogen, Carlsbad, CA, USA) was utilized as secondary antibody. Rabbit anti-P-p70S6K (Cell Signaling; 1:1000), rabbit anti-LC3 (Cell Signaling; 1:1000), mouse anti-LC3 (Nanotools; 1:100) and anti-4E-BP1 were from Cell Signaling Technology Inc (China).

**Determination MPO-DNA complexes levels in the supernatant**

MPO-DNA complexes were detected using a capture ELISA. Briefly, diluted supernatant were added to high-binding 96-well ELISA microplates (Greiner Bio-One) pretreated with capture anti-MPO (1:2000, Calbiochem, rabbit) antibody and blocked with 1% BSA/0.1% human serum albumin/PBS for 1-2 h. After overnight incubation and three washes with Tween20 in PBS, secondary peroxidase-labeled anti-DNA monoclonal antibody (Roche, Cat. No: 11774425001) was added for 30-60 min at RT according to the manufacturer’s instructions. The samples were washed three times with PBS per well and the peroxidase substrate (ABTS) of the kit (Roche, Cat. No: 11774425001) was added. The absorbance at 405 nm wavelength was measured using the Tecan microplate reader (Tecan Infinite M200) after 40 min incubation at 37 °C in the dark.

**Cell viability analysis**

Cell viability was observed by the trypan blue dye-exclusion assay and confirmed by MTT assay. Cells were plated on 96-well plates and incubated with various concentrations of ATO for the indicated times. A 10-μl aliquot of cell suspension was incubated with 10 μl 0.4% trypan blue solution for 5 minutes at room temperature. Viable and nonviable cells were defined based on absence and presence of intracellular trypan blue dye, respectively. Percentages were counted by hemacytometer. Cell viability % = viable cell numbers/total (viable+ dead) cell numbers ×100%.

**Immunoblot analysis**

Cells were solubilized at 4 °C in lysis buffer. The cell lysate (50 mg) was loaded onto a 12% sodium dodecyl sulfate-polyacrylamide gel, subjected to electrophoresis and transferred onto a nitrocellulose membrane. Blots were incubated with specific antibodies and washed, and proteins were visualized using the enhanced chemiluminescence system (Santa Cruz, Germany). The following antibodies were used: rabbit anti-P-p70S6K, rabbit anti-LC3, mouse anti-LC3, mouse anti-PML, rabbit anti-PML and anti-4E-BP.

**Determination of ROS generation**

The intracellular alterations of reactive oxygen species (ROS) were determined by measuring the oxidative conversion of cell-permeable 2’,7’-dichlorofluorescein diacetate (DCFH-DA) into fluorescent dichlorofluorescein (DCF) on a fluorospectrophotometer (F4000, Japan). In brief, NB4 cells with different treatments were collected, rinsed with D-Hank’s buffer and incubated with DCFH-DA (20 μM) at 37 °C for 20 min. Then the DCF fluorescence of 20,000 cells was detected by fluorospectrophotometer at an excitation wavelength of 488 nm and an emission wavelength of 535 nm. The incremental production of ROS was expressed as a percentage of control.

**Side population of NB4 cells**

Cells (1×10^6^ cells/ml) were incubated in prewarmed DMEM with 2% FCS (Invitrogen) containing freshly added Hoechst 33342 (Molecular Probes, Strasbourg, France) at a final concentration of 5 μg/ml for 100 minutes at 37 °C with intermittent mixing. In some experiments, cells were incubated with 50 μM verapamil (Sigma) for 20 min before Hoechst 33342 staining. At the end of the incubation, cells were cells were spun down at 4°C and resuspended in ice-cold Hank’s balanced salt solution (HBS) with 2% FCS and 10 mM HEPES buffer. Propidium iodide was added at a final concentration of 2 μg/ml for 5 minutes before FACS analysis, which allowed discrimination between dead and living cells. Samples were analyzed on a Becton Dickinson FACSAria. The Hoechst dye was excited with the 407 nm violet laser and its fluorescence measured with a 450/40 nm side population filter (Hoechst blue) and a 620 LPEFLP optical filter (Hoechst red). A 595 DCLP filter was used to separate the emission wavelengths.

**Experimental animals**

Experiments were carried out in the animal facility of the Animal Experimental Center of The Key Laboratory of Myocardial Ischemia in accordance with the Animal Care and Use Committee approved protocol. Forty-two male or female SCID mice, aged 5-7 weeks, were purchased from Beijing Vital River Laboratory Animal Technology Co., Ltd. The mice were housed in sterilized cages and provided with autoclaved water and a standard powdered rodent diet ad libitum. Experimental protocols were initiated after a 7-day acclimatization period.

**In vivo xenograft model^1^**

Xenograft model of APL was established by intravenous (i.v.) inoculation of exponentially growing NB4 cells (5x10^6^/mouse) in SCID mice. Our lab previously established a xenograft tumor model in SCID mice by using NB4 cell line. Tumors became visible at 14 days after NB4 cells were injected into mice. After 14 days of inoculation with NB4 cells, mice were randomly divided into three groups (six mice per group). The placebo group received the vehicle solution. Xenograft mice began to receive a treatment after 14 days of inoculation with NB4 cells when tumor was detected (appearance of typical illness: symptoms: wrinkled fur, depression, less activity, arch position, gait instability, cornering or circling, limb paralysis, thin, loss of appetite and multilymph node enlargement and specific cell surface markers’ detection in the peripheral blood cells).^1^

**Experimental design**

ATO was given to mice intraperitoneally at a concentration of 5 mg/kg starting on day 15 post injection of malignant cells and continued for 14 days. Rapamycin was started 3 days before ATO administration at a dose of 0.5 mg/kg and continued for 14 days at a dose of 0.25 mg/kg.^2^ Despite its oral bioavailability, rapamycin was administered intramuscularly to ensure stable blood levels. Before administration, rapamycin was suspended in a sterile solution containing 0.2% sodium carboxymethyl cellulose and 0.25% polysorbate-80. The placebo group received the vehicle solution.

**siRNA transfection**

NB4 cells were transiently transfected with Atg7 siRNA (no. 6604; Cell Signaling Technology, Danvers, MA, USA) at a concentration of 100 nM using Lipofectamine RNAiMAX Transfection Reagent (Life Technology, Grand Island, NY, USA), according to the manufacturer’s instruction (RNAiMAX Reverse Transfections Lipofectamine). The cells were also treated with a scrambled siRNA (no. 6568; Cell Signaling Technology) as a negative control. Seventy-two hours after transfection, cells were treated with the APL serum for 3 h. The silencing efficiency of Atg7 was determined by western blot of LC3 expression.

**Reference:**

1. [Valiuliene G](https://ssl.metstr.com/webfmrs/javascript:void(null)), [Stirblyte I](https://ssl.metstr.com/webfmrs/javascript:void(null)), [Cicenaite D](https://ssl.metstr.com/webfmrs/javascript:void(null)), [Kaupinis A](https://ssl.metstr.com/webfmrs/javascript:void(null)), [Valius M](https://ssl.metstr.com/webfmrs/javascript:void(null)), [Navakauskiene R](https://ssl.metstr.com/webfmrs/javascript:void(null)). Belinostat, a potent HDACi, exerts antileukaemic effect in human acute promyelocyticleukaemia cells via chromatin remodelling. *[J Cell Mol Med](https://ssl.metstr.com/webfmrs/javascript:void(null))* [2015;](https://ssl.metstr.com/webfmrs/javascript:void(null)) **[19](https://ssl.metstr.com/webfmrs/javascript:void(null))**: 1742-1755.
2. [Gallo R](https://ssl.metstr.com/webfmrs/javascript:void(null)), [Padurean A](https://ssl.metstr.com/webfmrs/javascript:void(null)), [Jayaraman T](https://ssl.metstr.com/webfmrs/javascript:void(null)), et al. Inhibition of Intimal Thickening After Balloon Angioplasty in Porcine Coronary Arteries by Targeting Regulators of the Cell Cycle. *Circulation* 1999; **99**: 2164-2170.
