# Supplementary material for: Arsenic trioxide promoting ETosis in acute promyelocytic leukemia through mTOR-regulated autophagy
Source: Cell Death Dis. 2018 Jan 23;9(2):75. doi: 10.1038/s41419-017-0018-3 (PMC5833714; doi:10.1038/s41419-017-0018-3)

**Supplementary Results:**

**Supplementary Figure S1**

**Comparison between** **ETosis% counted by DAPI/anti-histone-3 and** **lactadherin/PI staining**. Each circle represents one independent experiment done in duplicate, the solid line shows the hypothetical perfect agreement.


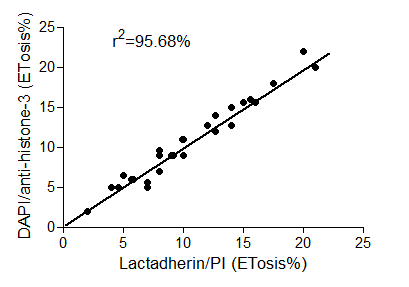


**Supplementary Figure S2**

NB4 cells were treated with 0, 0.1, 0.25, 0.5, 0.75, 1.0, or 2.0 μM ATO for 24, 48 and 72 hours. Representative PI-stained images of ET-releasing process: bubbling, budding, spread and extracellular DNA. Bars represent 5 μm.

extracellular

spread

budding

round

**Supplementary Figure S3**

NB4 cells were transfected with control siRNA (siCT) or siRNA specifically targeting *4E-BP1* (si*4E-BP1*), as indicated. Equal amounts of total cell lysates were analyzed by SDS-PAGE and immunoblotted with antibody against 4E-BP1 and compared to untreated control sample (upper panel). The same blot was then re-probed with anti-tublin antibody to control for protein loading (lower panel).


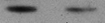

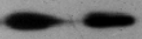


**si*4E-BP1***

**siCT**

**Tubulin**

**4E-BP1**

**Supplementary Figure S4**

**Reversal of the suppressive effects of ATO on ETosis by 4E-BP1 knockdown.** NB4 cells were transfected with either control siRNA or siRNA specifically targeting *4E-BP1* and subsequently incubated in the presence or absence of ATO. The percentage of ETosis and the concentration of MPO-DNA complexes were then measured (n = 5). **P* < 0.05 versus the absence of ATO, *^#^P* < 0.05 comparing the effects of ATO in the presence of *4E-BP1* siRNA versus control.


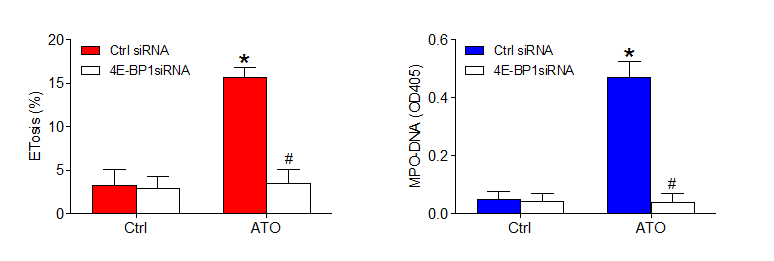


**Supplementary Figure S5**

**Inhibition or activation of autophagy with wortmannin and rapamycin did not alter basal ROS levels or ATO-induced ROS production**. NB4 cells were pretreated with DPI (10 μM, 4 hours) and cultured in the presence or absence of ATO (0.75 μM) for 48 hours. (A) NB4 cells were pretreated with vehicle (0.1% DMSO), rapamycin (10 nM) or wortmann for 30 min and then stimulated with ATO. ROS production was assessed by loading cells with the ROS sensor H2DCFDA (20 μM) followed by measuring the fluorescent intensity of DCF on a fluorescence plate reader. The incremental production of ROS was expressed as a percentage of control. **P* < 0.05 versus control, *^#^P* < 0.05 versus the absence of DPI. DPI, diphenyleneiodonium chloride.

**Supplementary Figure S6**

**ROS are required for enhanced ET release**. NB4 cells were stained with DAPI (blue) and anti-histone-3 (green). Immunostaining images showed ET-releasing cells (arrows) and that ET generation was abrogated by pretreatment with DPI. ETosis was evaluated by ET-releasing cells (%) and MPO-DNA complexes in the supernatant. NB4 cells in the absence of ATO were used as a control. All data shown are representative of mean ± SD (n = 6). **P* < 0.05 versus control, *^#^P* < 0.05 versus the absence of DPI. Bars represent 20 μm.


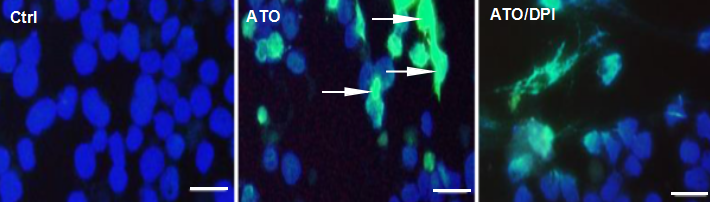


**Supplementary Figure S7**

**Effects of ASC and ATO on ETosis of resistant cell lines (NB4-R4 and NB4 ATO-R ).** ETosis induced by 1 μM ATO and increasing concentrations of rapamycin (0, 5, 10 and 20 nM) was evaluated at 48 h in NB4-R4 and NB4 ATO-R leukemia cell lines by lactadherin/PI staining. All data shown are representative of mean ± SD (n = 6). **p* < 0.05; ***p* < 0.001; ns: not significant.


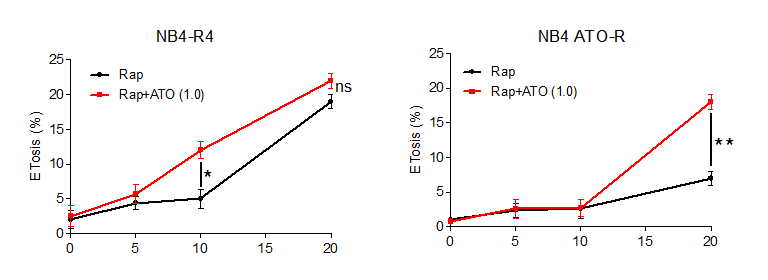

Supplement: Supplementary file 2 — Supplementary Results [file 41419_2017_18_MOESM2_ESM.docx]
